# Predictive validity of preschool screening tools for language and behavioural difficulties: A PRISMA systematic review

**DOI:** 10.1371/journal.pone.0211409

**Published:** 2019-02-04

**Authors:** Fiona Sim, Lucy Thompson, Louise Marryat, Nitish Ramparsad, Philip Wilson

**Affiliations:** 1 Centre for Rural Health, Centre for Health Sciences, University of Aberdeen, Inverness, United Kingdom; 2 Farr Institute/Scottish Collaboration for Public Health Research and Policy, University of Edinburgh, Edinburgh, United Kingdom; 3 Salvesen Mindroom Centre Research Centre for Learning Difficulties, University of Edinburgh, Edinburgh, United Kingdom; 4 Robertson Centre for Biostatistics, Institute of Health and Wellbeing, University of Glasgow, Glasgow, United Kingdom; Utah State University, UNITED STATES

## Abstract

**Background:**

Preschool screening for developmental difficulties is increasingly becoming part of routine health service provision and yet the scope and validity of tools used within these screening assessments is variable. The aim of this review is to report on the predictive validity of preschool screening tools for language and behaviour difficulties used in a community setting.

**Methods:**

Studies reporting the predictive validity of language or behaviour screening tools in the preschool years were identified through literature searches of Ovid Medline, Embase, EBSCO CINAHL, PsycInfo and ERIC. We selected peer-reviewed journal articles reporting the use of a screening tool for language or behaviour in a population-based sample of children aged 2–6 years of age, including a validated comparison diagnostic assessment and follow-up assessment for calculation of predictive validity.

**Results:**

A total of eleven eligible studies was identified. Six studies reported language screening tools, two reported behaviour screening tools and three reported combined language & behaviour screening tools. The Language Development Survey (LDS) administered at age 2 years achieved the best predictive validity performance of the language screening tools (sens 67%, spec 94%, NPV 88% and PPV 80%). The Strengths and Difficulties Questionnaire (SDQ) administered at age 4 years achieved the best predictive validity compared to other behaviour screening tools (Sens 31%, spec 93%, NPV 84% and PPV 52%). The SDQ and Sure Start Language Measure (SSLM) administered at 2.5 years achieved the best predictive validity of the combined language & behaviour assessments (sens 87%, spec 64%, NPV 97% and PPV 31). Predictive validity data and diagnostic odds ratios identified language screening tools as more effective and achieving higher sensitivity and positive predictive value than either behaviour or combined screening tools. Screening tools with combined behaviour and language assessments were more specific and achieved higher negative predictive value than individual language or behaviour screening tools. Parent-report screening tools for language achieved higher sensitivity, specificity and negative predictive value than direct child assessment.

**Conclusions:**

Universal screening tools for language and behaviour concerns in preschool aged children used in a community setting can demonstrate excellent predictive validity, particularly when they utilise a parent-report assessment. Incorporating these tools into routine child health surveillance could improve the rate of early identification of language and behavioural difficulties, enabling more informed referrals to specialist services and facilitating access to early intervention.

## Introduction

Developmental screening in the preschool years is increasingly attracting the attention of policy makers and clinicians, yet this remains a contentious area. Proponents cite the importance of moderate delays, which are harder to identify in community or primary care settings and yet carry pervasive effects into later childhood [1, 2], while opponents have raised concerns about costs and lack of robust screening instruments [3]. The aim of this comprehensive review is to report on the predictive validity of screening tools for language and behaviour difficulties utilised in a community preschool setting. Language and behaviour difficulties have been identified as key overlapping neurodevelopmental problems [4] which present in the preschool years and can predict poor psychiatric, educational and social outcomes into adolescence and adulthood [5, 6].

### Screening for language delay

Delayed language development can have a profound impact on the way in which a child views and interacts with the world. Language concerns identified in the preschool years often persist and can impact upon multiple domains of a child’s life in the early school years [7], into adolescence [5] and adulthood[6]. Particular problems associated with early language delay include learning difficulties [8], poorer health and behavioural outcomes [7] and unemployment in adulthood [9].

In the United States, prevalence of language delay, based on children aged 3 to 5 years receiving services for speech and language disabilities, was around 2.6% of the population [10] and data from a universal community surveillance of 2.5 year old children in Scotland estimated prevalence of between 3–8% of the population[4]. Depending on the definition and metric employed in quantifying language delay, this figure could be as high as 23% of preschool children experiencing delayed language development [11].

A Cochrane review conducted by Law and colleagues [12] found that there was insufficient evidence to merit the introduction of universal screening for speech and language delay but stressed that speech and language development remain a focus of routine child surveillance. Since then the Health for all Children Revised Fourth Edition (Hall 4) report, shaped government recommendations to incorporate surveillance or screening for speech and language disorders into routine primary care practice [13, 14], but implementation of this remains inconsistent [15, 16]. Widely used screening tools for language development include the Ages and Stages Questionnaire [17], the Language Development Survey [18] and the McArthur-Bates Communicative Development Inventory [19] but the majority are poorly validated.

### Screening for behaviour difficulties

The distinction between psychopathology and normal maturation is often indistinct in early childhood; behavioural patterns of aggression, non-compliance, hyperactivity and destructive behaviour may all be part of normal development until they are displayed at high levels indicating increased risk of continued behaviour problems [20]. This concept of a continuum of mental health has been particularly expressed in research demonstrating the common occurrence of features of autistic spectrum disorders in non-clinical populations of children [21]. Preschool behaviour problems have been associated with poorer outcomes in language and general development, health, behaviour and school life in the early school years [7] and adverse physical, mental health and forensic outcomes into adulthood [22–24]. Prevalence of preschool behavioural problems have been estimated at 4.8% in a Danish community sample [25], 8.6% in a German sample [25] and 8.8% in a Scottish sample [4].

As with screening for language delay, the implementation of standardized screening for behavioural concerns in the preschool years is variable. In the US, state laws often mandate that children are screened prior to school entry in order to gauge support needs but there is little consensus in how this is delivered [26]. In Scotland, Denmark, Finland, Norway and Sweden; child health policy explicitly aims to screen for problems in child development and each country has a focussed programme of child surveillance in place to meet this aim [27].

### Issues with preschool screening

Within the field of medicine, screening for preclinical disease is commonplace and highly successful in areas such as oncology and audiology [28, 29]. This success has not translated into the field of paediatric developmental screening, but with 60% of young people with developmental or mental health difficulties not being detected prior to school entry [30, 31] it is clear that our current detection methods are somewhat lacking. Due to the individual differences in developmental trajectory in the preschool years and the complexity in mental health screening more broadly, the implementation of routine screening is not a straightforward task. Criteria for population screening, outlined by Wilson and Jungner [32] are still pertinent in relation to availability of interventions and evidence of superior efficacy of early intervention [19]. Concerns relating to stigma [33]; lack of consensus on age at which to screen for developmental concerns as well as disagreement over diagnostic thresholds eliciting intervention [34]; combined with stretched primary care resources [3] have all contributed to a lack of clarity as to the best way to progress with universal screening programmes.

### Measuring validity of screening tools

Screening tools are designed to allocate the individuals being screened into one of two groups; those at risk of developing the condition and those who are not. Screening accuracy measures the association between risk group allocation and later diagnostic status (i.e. whether the individual has developed the condition or not). Statistically this is assessed by calculating the *sensitivity* (the proportion of true positives [TPs]); *specificity* (the proportion of true negatives [TNs]); *positive predictive value* (the proportion of those classified as at-risk who did develop the outcome [PPV]); and *negative predictive value* (the proportion classified as not at-risk in whom the outcome is absent [NPV]). For screening measures that are compatible with variable cut-off points, the trade-off between sensitivity and specificity can be analysed using a receiver operating curve (ROC), allowing for the identification of optimal cut-points [35].

Perhaps one of the foremost concerns relating to preschool developmental screening is a lack of well validated screening tools. While there are numerous studies demonstrating the construct and concurrent [36–38] validity of preschool screening tools, there is a dearth of evidence relating to the predictive validity of these tools when used within a community setting. Predictive validity is a key criterion in determining the efficacy of a screening tool as it ensures that the tool provides not just a snapshot of how a child is developing at a specific time point but also allows some insight into the progression of their development in subsequent years.

Having ascertained the prevalence and pervasiveness of language and behavioural difficulties formed during the preschool years, and outlined the case for (and concerns regarding) universal screening for these difficulties; the screening tools currently utilised in this population will now be reviewed and compared in terms of their validity in predicting child outcomes.

### Objectives

The aims of the current review were to:

- Report on the predictive validity of screening tools for language difficulties utilised in a community preschool setting
- Report on the predictive validity of screening tools for behaviour difficulties utilised in a community preschool setting

## Methods

### Protocol and registration

The protocol for this systematic review was registered with PROSPERO on the 28^th^ July 2017, registration number CRD42017072027.

### Eligibility criteria

Peer reviewed journal articles reporting the use of a screening tool for language or behaviour difficulties in a population-based sample of children aged 2–6 years of age, including a validated comparison diagnostic assessment and follow-up assessment for calculation of predictive validity (of which all data must be reported) were included. Complete inclusion and exclusion criteria are presented in Table 1.

**Table 1:** Complete inclusion and exclusion criteria.

Include	Exclude
Reported after 1946	Case-control study
Universal (community setting/whole population etc.) *Not high/low SES only*	High-risk groups; *Clinic referred*, *LAAC*, *low income*, *downs syndrome*, *preterm birth*, *concerns raised*
Studies involving a screening tool for language difficulties *Including dyslexia*	Studies based on populations of bilingual children
Studies involving a screening tool for behavioural difficulties *Including eating disorders*	Foreign language papers
Test designed to be used in (a) a primary health care setting and/or (b) in an educational setting by non-specialist staff for early identification, not diagnosis	Reported only concurrent validity/Construct validity/Internal consistency
All ethnicities (Unless the screening tool has been developed specifically for use in this population*)*	Retrospective study
Reported predictive validity *Sensitivity*, *specificity*, *NPV & PPV*	Intervention study
Prospective study	Book chapters/theses/conference abstracts
Screening for specific disorders (e.g. ASD, DBD) as long as it is based in the general population and not patient group	
Peer reviewed papers only	
Papers which compare a screening tool to a gold standard diagnostic assessment (e.g. DAWBA, Griffiths, Bayley, Reynell)	
Population of children aged 2-6years for initial assessment but follow-up anytime	
Clear criteria for defining language or behavioural difficulties based on cut-off scores on gold-standard norm-referenced tests or objectified clinical judgement	

Journal articles published in English before 28^th^ July 2017 were eligible for inclusion.

### Information sources

We searched Ovid Medline 1946 –March week 2 2017, Embase 1947 –present (updated daily), EBSCO CINAHL 1983–2017, PsycInfo 1914–2017 and ERIC 1959–2017.

Once the final sample of articles had been selected, the first author used the reference list of each of these articles as a secondary data source.

### Search

A search was carried out on the 28^th^ July 2017 using the following strategy:

1. child/ or child, preschool/
2. (child* or preschool* or kindergar?en*).tw
3. 1 or 2
4. Psychometrics/
5. (screening tools or assessment*).tw
6. 4 or 5
7. Child Development/
8. Neurodevelopmental disorders/ or developmental disabilities/
9. (language or communicat* or neurodevelopment* or development*).tw
10. 7 or 8 or 9
11. child behavior/ or problem behavior/
12. exp Social Behavior
13. “Disruptive, Impulse Control, and Conduct Disorders”/
14. Conduct Disorder/
15. (conduct or behavio?r).tw
16. 11 or 12 or 13 or 14 or 15
17. 3 and 6 and 10 and 16

### Study selection

**Fig 1:**
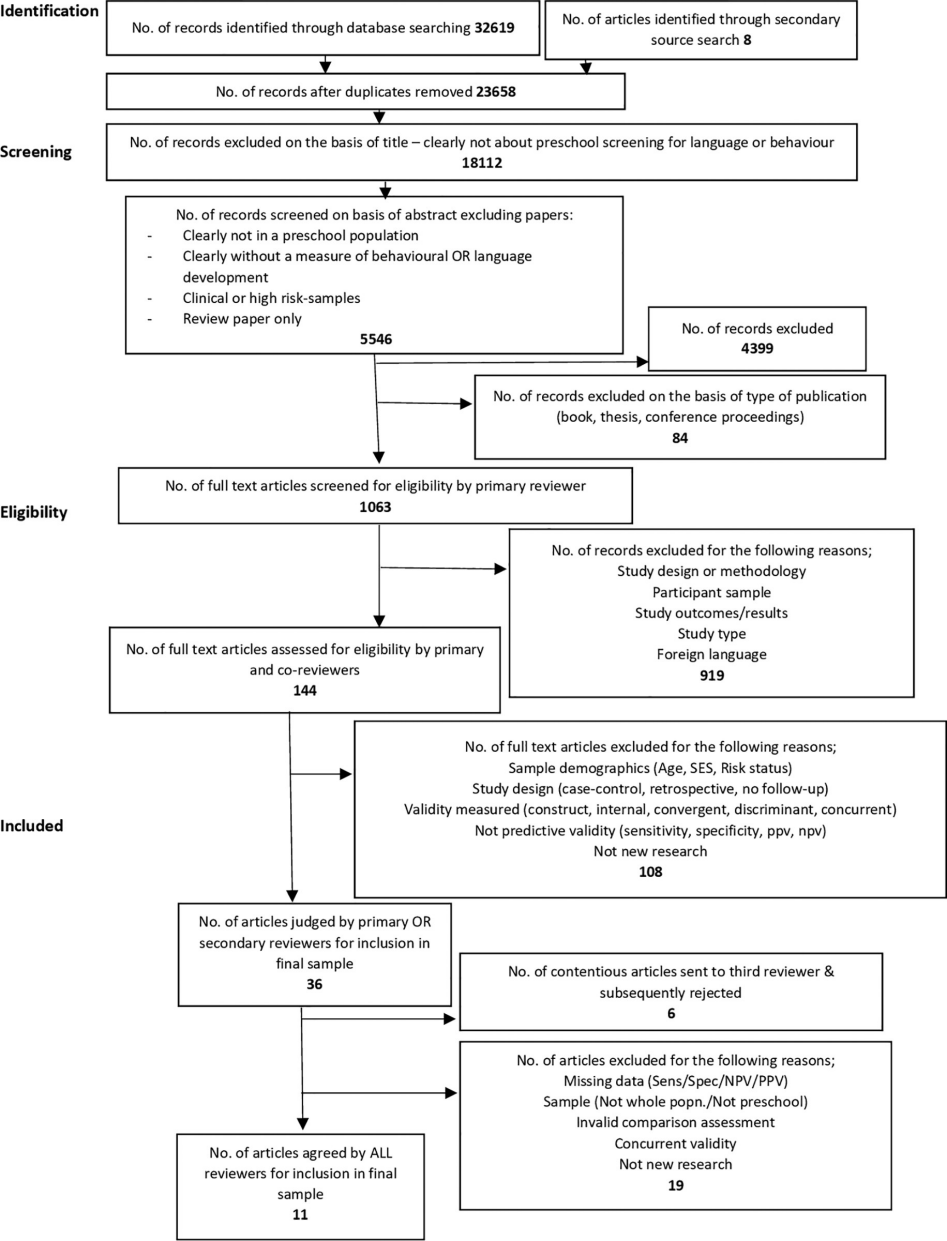
PRISMA diagram.

The study selection process is illustrated in Fig 1.

Screening:

In the first stage, papers were excluded based on their title, if they did not clearly report preschool screening for language or behaviour difficulties.

In the second stage of screening, papers were excluded on the basis of title and abstract, if they were not clearly:

- Reporting on preschool children aged 2–6 years
- Measuring language or behavioural development
- Utilising a population based sample

In addition; review papers, book chapters and conference proceedings were also excluded at this stage.

Full-text files were obtained for the remaining records.

Papers were rejected at this stage if they:

- Were not available in English
- Did not report original data
- Used a clinic referred or high risk sample
- Did not report on a distinct preschool population
- Did not include a validated assessment for comparison
- Did not include a follow-up assessment for calculation of predictive validity

All final sample papers were assessed by a second reviewer to reduce the risk of inclusion bias. Those papers whose inclusion was disputed by the first and second reviewers, were sent to a third reviewer and subsequently included or rejected.

Data extracted from eligible papers was tabulated and used in the qualitative synthesis.

### Data collection process

Data were collected onto a form developed by the first author, based on a form utilised by Law and colleagues in a large systematic review on screening for speech and language delay commissioned by the NHS R&D Health Technology Assessment programme[12]. For each paper, the first author completed the data collection form. As our analysis concerned only published data, we did not seek to obtain further data from authors.

### Data items

The variables extracted from each study are included in Supporting information (S1 Table).

### Risk of bias in individual studies

A Critical Appraisal Skills Programme (CASP) Diagnostic Checklist was completed for each study to document risk of bias. These data are reported qualitatively.

### Risk of bias across studies

Due to time and financial constraints, translators were not employed to assist in this review process. Papers published in any language other than English were therefore excluded. It is inevitable that this would introduce a degree of bias in the final sample of studies reported here.

### Diagnostic accuracy measures

The principal measure of diagnostic accuracy is the predictive validity of the screening tool compared with a validated diagnostic follow-up assessment. Primary outcome data are the sensitivity, specificity, negative predictive value (NPV) and positive predictive value (PPV). The area under the curve (AUC) resulting from receiver operating characteristic (ROC) analysis provides an estimate of the discriminative power of a diagnostic test and is reported if included in the study results.

### Quantitative synthesis of results

Based on the observed heterogeneity of results across the final sample of studies; random effects models of sensitivity and specificity data were generated from the best performing screening assessments for each individual study, and grouped based on whether they reported screening tools for language/behaviour/or a combined language and behaviour screening tool. Heterogeneity was assessed with the I-squared statistic (i.e. the percentage of variation across studies that is due to heterogeneity rather than chance). In order to provide an overall measure of the effectiveness of the screening tests, a diagnostic odds ratio was calculated based on the best performing screening test reported in each of the final studies. The diagnostic odds ratio (DOR) is a global measure for diagnostic test accuracy that is independent of prevalence, and represents the ratio of the odds of positivity in subjects with disease relative to the odds in subjects without disease [39].

### Qualitative synthesis of results

Due to the heterogeneity of the studies, data were synthesized qualitatively by comparing predictive validity statistics across studies and exploring age and respondent effects on predictive performance. Eligible papers are assigned to one of three categories; studies reporting language screening tools; studies reporting behavioural screening tools; and studies reporting combined language & behavioural screening tools. Within each category, studies are reported in descending order of overall predictive validity performance.

## Results

### Study selection

The study selection process is illustrated in Fig 1. The PRISMA checklist is included in Supporting information (S1 Fig).

Each of the articles selected for the final sample was reviewed by two independent reviewers and when those reviewers disagreed, a third independent reviewer was consulted.

### Study characteristics

Study characteristics are presented in Table 2.

**Table 2:** Characteristics of included studies.

REFERENCE	METHODS	SCREENING PROCEDURE	PARTICIPANT CHARACTERISTICS	DIAGNOSTIC CRITERIA SCREEN	DIAGNOSTIC CRITERIA FOLLOW-UP	RESULTS
						Primary
	• *Study design* • *Screen sample size* • *Follow-up sample size* • *Language* • *Analysis*	• *Name of screen test* • *Areas tested* • *Administration time* • *Screener* • *Respondent* • *Name of follow-up test*	• *Total number* • *Age at first assessment* • *Age at FU assessment* • *Sex* • *Ethnicity (inc*. *language)* • *SES*	• *Type of delay/disorder* • *Cut-off/diagnostic criteria* • *Rationale for criteria*	• *Type of delay/disorder* • *Cut-off/diagnostic criteria* • *Rationale for criteria*	• *Sensitivity* • *Specificity* • *NPV* • *PPV*
*Cadman*, *D*., *Walter*, *S*. *D*., *Chambers*, *L*. *W*., *Ferguson*, *R*., *Szatmari*, *P*., *Johnson*, *N*., *McNamee*, *J*. *“Predicting problems in school performance from preschool health*, *developmental and behavioural assessments” Canadian Medical Association Journal 1988*, *139*, *1*.	• Prospective cohort study • N = 2761 • N = 1999 • English • Predictive accuracy; logistic regression analysis; Receiver Operating Curve (ROC) analysis	• Denver Developmental Screening Test (DDST); health, behaviour & neurodevelopmental histories; vision & hearing screening tests • Language; fine motor; gross motor; personal-social; behaviour & neurodevelopmental history; vision & hearing • FU: Teacher reported learning problems; placement in special classes; Gates-MacGinitie reading test	• 1999 • 47–62 months • 83–98 months	• Developmental delay • Below, at or above median DDST score for study population	• School problem • Child still in grade 1 because of academic problems; child in a special education class; teacher rated learning problem • Reading problem • Lowest 10^th^ percentile on Gates-MacGinitie reading test	• DDST alone • Sens 6% Spec 99% • NPV N/R PPV 73% • DDST and Health, development & behavioural history • 20^th^ centile • Sens 44% Spec 85% • NPV 87% PPV 41% • 10^th^ centile • Sens 27% Spec 93% • NPV 85% PPV 50%
*Cadman*, *D*., *Chambers*, *L*. *W*., *Walter*, *S*. *D*., *Feldman*, *W*., *Smith*, *K*., *Ferguson*, *R*. *(1984) “The Usefulness of the Denver Developmental Screening Test to Predict Kindergarten Problems in a General Community Population” American Journal of Public Health 74*: *1093–1097*	• Prospective community cohort study • N = 2569 • N = 2443 (95.1%) • Prevalence estimates, predictive validity (sens, spec, NPV, PPV)	• Denver Developmental Screening Test (DDST) • Gross motor, language, fine motor, personal-social development • Direct assessment of child • FU: Teacher rated global ratings of child academic and learning abilities, classroom behaviour and amount of special attention required in the classroom	• N = 2443 • 47–62 months • 61–76 months • Male N = 1259 • Female N = 1310	• Developmental disability • All children who received positive screen were re-tested with the DDST–those who received an abnormal, questionable or untestable result on both tests were classified as screen positive	• School problems • Teacher global ratings of child academic and learning abilities, classroom behaviour and amount of special attention required in the classroom, Referral to special education services	• Predicting learning difficulties • Sens 6% 95%CI (4–8) • Spec 99% 95%CI (99–99) • NPV 84% 95%CI (83–86) • PPV 55% 95%CI (39–70) • Predicting behaviour problems • Sens 5% 95%CI (3–8) • Spec 99% 95%CI (98–99) • NPV 89% 95%CI (89–90) • PPV 31% 95%CI(18–47) • Predicting special attention in classroom • Sens 21% 95%CI (21–22) • Spec 5% 95%CI (3–7) • NPV 79% 95%CI (77–80) • PPV 62% 95%CI (46–76) • Predicting specialist referral • Sens 10% 95%CI (6–14) • Spec 99% 95%CI (99–99) • NPV 93% 95%CI (92–94) • PPV 45% 95%CI (30–61) • *Secondary results: User perspectives • *Trained public health nurses can reliably administer and score the DDST*
Dale, P.S., Price, T.S., Bishop, DVM & Plomin, R. “Outcomes of early language delay: I. Predicting persistent and transient language difficulties at 3 and 4 years” American Speech-Language-Hearing Association 2003, 46.3	• Longitudinal birth cohort study • N = 8,386 • N at 3yrs = 7,808 (93.1%) • N at 4yrs = 6,660 (79.4%) • English • Relative risk ratios, logistic regression, predictive validity (sens, spec, NPV, PPV)	• MacArthur Communicative Development Inventory: UK Short Form (MCDI:UKSF) • 100 word list, 12 item grammar scale, 5 items from original MCDI combined to give a 10-point “Displaced reference scale” • Parent Report of Children’s Abilities (PARCA) • Vocabulary, grammar, contextual language, nonverbal ability • Parent report • 3yr FU: 45 words from MCDI 100 word list & 55 new words; Displaced Reference Scale; Abstract Language Scale; Parental language concerns; Communicative Abnormality Scale • 4yr FU: MCDI inc. 48 new words; Grammar Rating Scale; Abstract Language Scale; Parental language concerns; Communicative Abnormality Scale	• N = 8,386 • 2 years • 3 or 4 years	• Early language delay • Vocabulary score of 15 or less (10^th^ centile) • To obtain an adequate sample size the authors employed a less stringent cut-off than previous research	• Persistent language difficulties • Scores on 2 of 3 language measures at or below 15^th^ centile • At 3 years: raw scores <33 for vocabulary, 2 for grammar and 5 for abstract language • At 4 years: <29 for vocabulary, 6 for grammar, 8 for abstract language	• 10^th^ centile cut-off • Sens: 3yr 38.5% 4yr 44.6% • Spec: 3yr 76.2% 4yr 80.5% • NPV: 3yr 61.1% 4yr 67.7% • PPV: 3yr 56.1% 4yr 61.4% • 5^th^ centile cut-off • Sens: 3yr 50.0% 4yr 63.9% • Spec: 3yr 67.3% 4yr 70.0% • NPV: 3yr 60.5% 4yr 68.3% • PPV: 3yr 58.1% 4yr 65.6%
**Study 1:** *Girio-Herrera*, *E*., *Dvorsky*, *M*.*R*., *Sarno Owens*, *J*. *(2015) “Mental health screening in kindergarten youth*: *A multistudy examination of the concurrent and diagnostic validity of the impairment rating scale” Psychological Assessment 2015*, *27*:*1*.	• Multi-study, prospective cohort • N = 568 • N = 568 (100%) • Concurrent and diagnostic validity by examining within- and between-rater bivariate correlations and AUC statistics	• The Impairment Rating Scale (IRS) • BASC-2 parent report • Relations with peers, relations with teachers/parents (respondent dependant), relations with siblings, academic progress, self-esteem, classroom/family functioning (respondent dependant), and overall impairment • <5mins • Parent & teacher • BASC-2 teacher report	• N = 568 • Mean age 5.48 years • FU 8–12 weeks later • 46.8% male 53.2% female • 95.1% Caucasian (2.8% classified as other and less than 1% as African • American, Hispanic, Asian, and American Indian/Alaskan Native) • Middle and lower SES	• Risk for social, emotional and behavioural problems • Overall impairment IRS efficiency statistics for scores of 2,3 and 4 were examined • Cut-off of 3 or higher identified in previous research. 2,3 & 4 were examined for efficiency	• BASC-2 BESS teacher report • T-score of 60 or greater on either the Externalising Problems or Internalising Problems Composites or a T-score of 40 or lower on the Adaptive Skills Composite	• Parent IRS identifying teacher BASC-2 BESS • Sens: • cut-off 2: 15% • cut-off 3: 9% • cut-off 4: 5% • Spec: • cut-off 2: 90% • cut-off 3: 95% • cut-off 4: 98% • NPV: • cut-off 2: 80% • cut-off 3: 80% • cut-off 4: 80% • PPV: • Cut-off 2: 28% • Cut-off 3: 32% • Cut-off 4: 37% • AUC .53, SE .03 • 95%CI (.47-.59)
**Study 2:** *Girio-Herrera*, *E*., *Dvorsky*, *M*.*R*., *Sarno Owens*, *J*. *(2015) “Mental health screening in kindergarten youth*: *A multistudy examination of the concurrent and diagnostic validity of the impairment rating scale” Psychological Assessment 2015*, *27*:*1*.	• Multi-study, prospective cohort • N = 242 • N = 242 (100%) • Concurrent and diagnostic validity by examining within- and between-rater bivariate correlations and AUC statistics	• The Impairment Rating Scale (IRS) • BASC-2 parent report • Relations with peers, relations with teachers/parents (respondent dependant), relations with siblings, academic progress, self-esteem, classroom/family functioning (respondent dependant), and overall impairment • <5mins • Parent & teacher • BASC-2 BESS teacher report	• N = 242 • Mean age 5.61years • FU 2-6months later • 50.8% male 49.2% female • 95.5% Caucasian (2.1.% classified as Hispanic; less than 1% • African American, Asian, and American Indian/Alaskan Native)	• Risk for social, emotional and behavioural problems • Overall impairment IRS efficiency statistics for scores of 2,3 and 4 were examined • Cut-off of 3 or higher identified in previous research. 2,3 & 4 were examined for efficiency	• BASC-2 BESS behavioural and emotional problems screen • T-score of 61 or greater	• Parent IRS identifying teacher BESS • Sens: • Cut-off 2: 29% • Cut-off 3: 17% • Cut-off 4: 8% • Spec: • Cut-off 2: 91% • Cut-off 3: 95% • Cut-off 4: 97% • NPV: • Cut-off 2: 92% • Cut-off 3: 90% • Cut-off 4: 90% • PPV: • Cut-off 2: 29% • Cut-off 3: 28% • Cut-off 4: 22% • AUC .66, SE .07 • 95%CI (.53-.78)
*Missall*, *K*., *Reschly*, *A*., *Betts*, *J*., *McConnell*, *S*., *Heistad*, *D*., *Pickart*, *M*., *Sheran*, *C*. *and Marston*, *D*. *(2007) “Examination of the Predictive Validity of Preschool Early Literacy Skills” School Psychology Review; 36; 3*.	• Longitudinal cohort study • N = 110 • FU N = 88 (80%) • General latent variable modelling, multiple regression models, logistic regression model	• Early Literacy Individual Growth and Development Indicators EL-IGDI’s • Early literacy skills; picture naming, rhyming and alliteration • 10mins • Reading–Curriculum-based measurement (R-CBM)	• N = 116 • 4 years • FU 6 years • Females 54.5% Males 45.5% • 40% African American, 34% European American, 10% Asian American, 10% American Indian, and about 6% Hispanic American • 58% of the students were eligible for free or reduced-price lunch	• Early literacy difficulties • Picture naming subscale fail	• R-CBM cut-off 60 words per minute	• EL-IGDIs predicting R-CBM 60 word cut-off • Sens: 64% • Spec: 81% • NPV: 72% • PPV: 74%
*Owens*, *J*. *S*., *Storer*, *J*., *Holdaway*, *A*. *S*., *Serrano*, *V*. *J*., *Watabe*, *Y*., *Himawan*, *L*. *K*., *Krelko*, *R*. *E*., *Vause*, *K*. *J*., *Girio-Herrera*, *E*. *& Andrews*, *N*. *(2015) “Screening for Social*, *Emotional*, *and Behavioral Problems at Kindergarten Entry*: *Utility and Incremental Validity of Parent Report” School Psychology Review 44; 1*	• Prospective population cohort • N = 252 • FU N = 252 (100%) • Receiver operating curve (ROC) analysis, predictive validity	• Disruptive Behaviour Disorders (DBD) rating scale • Strengths and Difficulties Questionnaire (SDQ) • Disruptive behaviour disorders, socio-emotional functioning • Parent • BASC-2 BESS-Teacher Rating	• 252 • 4.87 years • FU 6 months later • 50.4% male 49.6% female • 94.8% white	• Social, emotional and behavioural disorders • DBD rating scale average score ≥1 denotes at-risk status • SDQ recommended cut scores (www.sdqinfo.org)	• BASC-2 BESS-teacher version • Internalizing, externalizing and adaptive behaviour problems • Cut score for age-based *t* score of 61 or higher	• SDQ behaviour problems • Sens: • Cut-off 2: 58% • Cut-off 3: 46% • Cut-off 4: 31% • Spec: • Cut-off 2: 68% • Cut-off 3: 83% • Cut-off 4: 93% • NPV: • Cut-off 2: 87% • Cut-off 3: 86% • Cut-off 4: 84% • PPV: • Cut-off 2: 31% • Cut-off 3: 40% • Cut-off 4: 52% • AUC: • .68 95%CI [.55, .80] • SDQ emotional problems • Sens: • Cut-off 1: 58% • Cut-off 2: 35% • Cut-off 3: 23% • Spec: • Cut-off 1: 39% • Cut-off 2: 65% • Cut-off 3: 82% • NPV: • Cut-off 1: 79% • Cut-off 2: 80% • Cut-off 3: 81% • PPV: • Cut-off 1: 19% • Cut-off 2: 20% • Cut-off 3: 25% • AUC: • .50 95%CI [.37, .62] • DBD hyperactivity-impulsivity • Sens: • Cut-off 0.5: 73% • Cut-off 1: 54% • Spec: • Cut-off 0.5: 49% • Cut-off 1: 79% • NPV: • Cut-off 0.5: 88% • Cut-off 1: 87% • PPV: • Cut-off 0.5: 26% • Cut-off 1: 39% • AUC: • .68 95%CI [.55, .80] • DBD oppositional defiant • Sens: • Cut-off 0.5: 50% • Cut-off 1: 31% • Spec: • Cut-off 0.5: 67% • Cut-off 1: 91% • NPV: • Cut-off 0.5: 84% • Cut-off 1: 84% • PPV: • Cut-off 0.5: 27% • Cut-off 1: 47% • AUC: • .63 95%CI [.51, .75] • *Secondary results: user perspectives • *Informal interviews with school staff suggest that screening reports were minimally and inconsistently used across teachers*
*Rescorla*, *L*. *& Alley*, *A*. *(2001) “Validation of the Language Development Survey (LDS)*: *A Parent Report Tool for Identifying Language Delay in Toddlers” Journal of Speech*, *Language*, *and Hearing Research*. *44*.*2*.	• Epidemiological, prospective cohort • N = 422 • FU N = 66 (15.6%) • Correlational analysis and odds ratios	• Language Development Survey (LDS) • 10mins • Parent • Reynell receptive and expressive language scales	• 422 • Mean 24.7months • FU mean 25.2 months • 50% male and female • Majority white • 81% middle- to upper-middle class (Hollingshead social class I and II)	• Expressive language delay • Delay 1 cutoff: <30 words AND no word combinations • Delay 2 cutoff: <30 words OR no word combinations • Delay 3 cutoff: <50 words OR no word combinations	• Expressive language delay • Reynell Z-score less than or equal to -1.25 (10^th^ percentile)	• Sens: • Delay1: 67% 2: 89% 3: 94% • Spec: • Delay 1: 94% 2: 77% 3: 67% • NPV: • Delay 1: 88% 2: 95% 3: 97% • PPV: • Delay 1: 80% 2: 59% 3: 52%
*Sachse*, *S*., *Von Suchodoletz*, *W*. *2008 “Early identification of language delay by direct assessment or parent report*?*” Journal of Developmental Pediatrics 29*:*34–41*.	• Prospective cohort study • N = 1056 • FU N = 102 (9.66%) • German • Descriptive statistics, concurrent validity, predictive validity	• MacArthur Communicative Development Inventory (MCDI) Toddler form (ELFRA-2) • Sprachentwicklungstest fur zweijahrige kinder (2.0–2.11) SETK-2, noverbal subscale of the Munchener Funktionelle Entwicklungsdiagnostik, hearing screen ECHO-SCREEN Plus-T • Productive vocabulary, syntax and morphology • Parent report • Sprachentwicklungstest fur zweijahrige kinder 3/5 (SETK-3/5)	• N = 102 • 24 months • FU mean age 37 months • Monolingual german	• Late talking (LT) toddlers • Productive vocabulary <50 words or 50–80 words • Syntax score <7, Morphology score <2 • Followed test instructions	• Language delay • 1SD below the mean on one of three subscales of SETK-3/5	• Sens: 61% • Spec: 94% • NPV: 95% • PPV: 56% • *secondary results: user perspectives • Accuracy of parent report dependent on mothers education level: • Vocabulary and word production scores tended to be lower in toddlers with less educated mothers–but differences were not significant
*Sim*, *F*., *Haig*, *C*., *O’Dowd*, *J*., *Thompson*, *L*., *Law*, *J*., *McConnachie*, *A*., *Gillberg*, *C*., *Wilson*, *P*. *(2015) “Development of a triage tool for neurodevelopmental risk in children aged 30 months” Research in Developmental Disabilities 45–46; 69–82*.	• Prospective cohort study • N = 486 • FU N = 103 (21.19%) • English • Receiver operating curve (ROC) analysis for optimised cut points • Predictive validity • Non parametric bootstrapping to produce confidence intervals	• Sure Start Language Measure (SSLM) • Strengths and Difficulties Questionnaire (SDQ) • Vocabulary, socio-emotional development • 15mins • Parent • Development and Wellbeing Assessment (DAWBA) • Griffiths Mental Development Scale-Extended Revised (GMDS-ER) • New Reynell Developmental Language Scale (NRDLS)	• N = 103 • 30months • FU mean 47.5months • 55% male 45% female • 41% living in most deprived quintile (Scottish Index of Multiple Deprivation)	• Language delay • Socio-emotional difficulties • <32 words on SSLM • >8 Total Difficulties Score SDQ • ROC curve analysis of optimal screen performance	• ICD-10 Psychiatric diagnosis from DAWBA • Language disorder: Comprehension or production scores 2SD below mean NRDLS • Global developmental delay: General performance 2SD below mean GMDS	• Sens: 87% 95%CI (76–96) • Spec: 64% 95%CI (59–71) • NPV: 97% 95%CI (94–99) • PPV: 31% 95%CI (23–39) • SSLM—NRDLS (AUC .905) SSLM—GMDS (AUC .983) • SDQ–DAWBA (AUC .821)
*Stott*, *C*. *M*., *Merricks*, *M*. *J*., *Bolton*, *P*. *F*., *Goodyer*, *I*. *M*. *(2002) “Screening for speech and language disorders*: *the reliability*, *validity and accuracy of the General Language Screen” International Journal of Language & Communication Disorders*, *37*:*2*, *133–151*.	• Longitudinal epidemiological study • N = 1936 • FU N = 254 (13.12%) 45mths • FU N = 218 (11.26%) 8yrs • Content validity, criterion validity, construct validity, predictive validity, Receiver Operating Curve (ROC) analysis, factor analysis	• General Language Screen (GLS) • Developmental Profile II (DPII) • Receptive and expressive language • Parent • 45 months: Edinburgh Articulation Test (EAT), Reynell Developmental Language Scales (RDLS), British Picture Vocabulary Scales (BPVS). • 8 years: Clinical Evaluation of Language Fundamentals–Revised (CELF-R)	• N = 254 at 45 months • N = 218 at 8 years • 36 months • FU 45 months and 8 years	• Speech/language difficulties • Parent endorsement of any one of the 11 speech/language-related GLS items OR any two of the 11 items	• Language function • 2SD below the mean on any one of the Edinburgh Articulation Test (EAT), Reynell Developmental Language Scales (RDLS), British Picture Vocabulary Scales (BPVS), Clinical Evaluation of Language Fundamentals–Revised (CELF-R)	• 2/11 GLS items endorsed • Sens: • 45mths:67.4% • 8yrs: 60.0% • Spec: • 45mths: 68.2% • 8yrs: 67.4% • NPV: • 45mths: 90.6% • 8yrs: 91.3% • PPV: • 45mths: 31.5% • 8yrs: 22.8% • AUC • 45mths: .77 • 8yrs: .68 • 1/11 GLS items endorsed • Sens: • 45mths: 97.7% • 8yrs: 90% • Spec: • 45mths: 35.9% • 8yrs: 31% • NPV: • 45mths: 98.6% • 8yrs: 95.1% • PPV: • 45mths: 24.9% • 8yrs: 17.3%
*Wilson*, *B*., *Lonigan*, *C*. *J*. *(2010) “Identifying preschool children at risk of later reading difficulties*: *Evaluation of two emergent literacy screening tools” Journal of Learning Disabilities 43(1) 62–76*	• Prospective cohort study • N = 199 • FU N = 176 (88.44%) • Descriptive statistics & correlations between time 1 & time 2 measures • Receiver Operating Curve (ROC) analysis	• Get Ready to Read! Screening Tool (GRTR) • Individual Growth and Development Indicators (IGDIs) • GRTR–print knowledge and phonological awareness • IGDIs–expressive communication, adaptive ability, motor control, social ability and cognition • Direct child assessment • Test of Preschool Early Literacy (TOPEL)	• N = 199 • 48.55 months • FU 3 months later • Male 61% Female 39% • 52% African American; 9% other	• Reading difficulties • TOPEL standard score cutoff of 90 (26^th^ percentile) for all three subtests • In choosing the 25^th^ percentile the goal was to identify a group of children performing at the lower end of the distribution of emergent literacy skills and therefore those who were more likely candidates for additional assessment/intervention than those scoring in higher percentiles	• TOPEL–print knowledge, definitional vocabulary, phonological awareness • Standard score cutoff of 90 (26^th^ percentile)	• GRTR predicting TOPEL ELI • Sens: 90% Spec: 69% • NPV: 38% PPV: 97% • AUC .86 • IGDI’s predicting TOPEL ELI • Sens: 93% Spec: 38% • NPV: 24% PPV: 97% • AUC: .73 • GRTR predicting TOPEL PK • Sens: 92% Spec: 56% • NPV: 35% PPV: 96% • AUC: .84 • IGDI’s predicting TOPEL PK • SENS: 94% Spec: 40% • NPV: 29% PPV: 97% • AUC: .76 • GRTR predicting TOPEL DV • Sens: 95% Spec: 15% • NPV: 13% PPV: 96% • AUC: .75 • IGDI’s predicting TOPEL DV • Sens: 95% Spec: 6% • NPV: 11% PPV: 90% • AUC: .71 • GRTR predicting TOPEL PA • Sens: 93% Spec: 23% • NPV: 36% PPV: 87% • AUC: .68 • IGDI’s predicting TOPEL PA • Sens: 93% Spec: 13% • NPV: 33% PPV: 79% • AUC: .64

Five studies failed to meet inclusion criteria for the final sample on the basis of missing elements of predictive validity data but did meet all other inclusion criteria. These studies are mentioned in a separate section of the results and study characteristics are presented in Table 3.

**Table 3:** Studies reporting screening tools failing to meet full inclusion criteria.

REFERENCE	METHODS	SCREENING PROCEDURE	PARTICIPANT CHARACTERISTICS	DIAGNOSTIC CRITERIA SCREEN	DIAGNOSTIC CRITERIA FOLLOW-UP	RESULTS
						Primary
	• *Study design* • *Screen sample size* • *Follow-up sample size* • *Language* • *Analysis*	• *Name of screen test* • *Areas tested* • *Setting* • *Administration time* • *Screener* • *Respondent* • *Name of follow-up test*	• *Total number complete* • *Age at first assessment* • *Age at FU assessment* • *Sex* • *Ethnicity (inc*. *language)* • *SES*	• *Type of delay/disorder* • *Cut-off/diagnostic criteria* • *Rationale for criteria*	• *Type of delay/disorder* • *Cut-off/diagnostic criteria* • *Rationale for criteria*	• *Sensitivity* • *Specificity* • *NPV* • *PPV*
*de Koning*, *H*.*J*., *de Ridder-Sluiter*, *J*.*G*., *van Agt*, *H*.*M*.*E*., *Reep-van den Bergh*, *C*.*M*.*M*., *van der Stege*, *H*.*A*., *Korfage*, *I*.*J*., *Polder*, *J*.*J*. *& van der Maas*, *P*.*J*. *(2004) “A cluster-randomised trial of screening for language disorders in toddlers” Journal of Medical Screening*, *2004*, *11*:*3*.	• Cluster-randomised screening trial • Longitudinal • N = 3,147 • N = 3,685 • Logistic regression analysis	• VTO Language Screening Instrument (VTO:LSI) • Language production, comprehension & interaction • 5mins • Parent • Specialist referral information; Dutch Parent Language Checklist (PLC); the LSI (age 3-4yrs); the LSI parents questionnaire (PQ); and Van Wiechen items	• N = 3,147 • 18 and 24 months • 36 months	• Language delay • Final summed score of both VTO LSI ≤2	• Specialist service referral information; Expert panel diagnosis; Language delay measured by the Dutch Parent Language Checklist (PLC); the LSI (age 3-4yrs); the LSI parents questionnaire (PQ); and Van Wiechen items	**• Specialist referral;** • Sens 52% Spec N/R • NPV N/R PPV 55% • **Parent report:** • Sens 24% Spec 97–98% • NPV N/R PPV N/R
Eadie, P., Nguyen, C., Carlin, J., Bavin, E., Bretherton, L., and Reilly, S. (2014) “Stability of language performance at 4 and 5 years: measurement and participant variability” International Journal of Language and Communication Disorders 49: 2, 215–227.	• Longitudinal cohort study • N = 1560 • N = 945 (60.58%) • English • Pearson correlation, Odds rations, predictive accuracy(sensitivity and specificity), Bland-Altman plots	• Clinical Evaluation of Language Fundamentals (CELF-P2) • Receptive language: Sentence structure, concepts and following directions, basic concepts • Expressive language: word structure, expressive vocabulary, recalling sentences • Direct child assessment • Clinical Evaluation of Language Fundamentals (CELF-4)	• N = 945 • 4.13 years • 5.15 years • 51.1% female • 98% English speaking background • Mean SEIFA index of disadvantage 1044 (SD 52)	• Language Impairment • 1.25SD below the mean	• Language Impairment: performance at least 1.25SD below the mean • Receptive: Sentence structure, Concepts and following directions and word classes • Expressive: word structure, recalling sentences, formulated sentences	• **CELF-P2–1.25SD** • Sens 64% Spec 92.9% • NPV N/R PPV N/R • **CELF-P2 -2SD** • Sens 42.1% Spec 98.6% • NPV N/R PPV N/R
*Fowler*, *M*., *G*. *and Cross*, *A*., *W*. *(1986) “Preschool risk factors as predictors of early school performance” Developmental and Behavioural Pediatrics Vol 7*. *No*.*4*	• Prospective cohort study • N = 210 • N = 176 (84%)	• Sprigle School Readiness Test (SSRT), Beery Test of Visual Integration (VMI), Risk Index of School Capability (RISC), Demographic questionnaire, likert rating of attention • Cognitive skills, visual motor skills, language, attention • 10-12mins SSRT, 3mins VMI • Direct child assessment & parent • Grade failure	• N = 176 • 55months • 79-103months • 61% white	• Cognitive delay, visual motor impairment, academic potential • RISC score ≥7(0–11) • SSRST cutoff score of 10 • VMI cutoff of ≥1 SD below group mean	• Grade failure	• **RISC ≥7** • Sens: 96% Spec: 33% • NPV: N/R PPV 98% • **RISC ≥5** • Sens: 71% Spec: 78% • NPV: N/R PPV: 39% • **RISC≥3** • Sens: 36% Spec: 97% • NPV: N/R PPV: 71% • **SSRST:** • PPV: 35% • **VMI:** • PPV 38%
*Westerlund*, *M*. *1995 “Predictive power of a phonological screening test at four years of age in relation to later linguistic ability” Scandinavian Journal of Logopedics and Phoniatrics*, *20*: *60–76*	• Prospective cohort study • N = 1658 • N = 1451 (87.5%) school start • N = 1328 (80.1%) grade 3 • Prevalence analysis, predictive validity; sens; spec; NPV; PPV	• Uppsala screening test inc. parent report language questionnaire • Receptive and expressive language • Direct child assessment and parent report • Phonological assessment by speech therapist • Reading comprehension test	• N = 1328 at grade 3 • 4 years • School start • Age 4: 869 boys 776 girls • Social class 1(high): 428 Social class 2: 643 • Social class 3(low): 353	• Language impairment • No impairment; Slight; Moderate; Severe	- *Phonological assessment by speech therapist at school start* • *No impairment (including lisping)*, *slight* ***(/s/*** *and/or /r/ mistakes)*, *moderate (other phonological problems hardly influencing understandability) and severe impairment (significant problems considerably influencing* • *understandability)*. - Reading comprehension test in grade 3 • *Pupils with scores 1–4 are defined as poor performers and those with scores* ***8*** *and* ***9*** *as good performers*. • - *School placement and grade in school the year after the children had reached the age of compulsory school start*	• 4yr screen & speech therapist assessment at school start • **Severe** • Sens 12% Spec 99% • NPV N/R PPV 43% • **Moderate to severe** • Sens 48% Spec 88% • NPV N/R PPV 19% • **Slight to severe** • Sens 71% Spec 69% • NPV N/R PPV 12% • **4yr screen & grade 3 assessment** • **Moderate to severe** • Sens 21% Spec 88% • NPV N/R PPV 29%
*Westerlund, M. and Sundelin, C. (2000) “Can severe language disability be identified in three-year-olds? Evaluation of a routine screening procedure” Acta Paediatrica 89: 94–100*	• Prospective cohort study • N = 2359 • N = 2237 (94.83%) • Swedish • Significance tests were performed using the chi-squared statistic and Fisher’s exact test, when expected counts were less than 5. The predictability of the 3-y screening was expressed in terms of sensitivity, specificity, positive predictive value and risk ratio.	• Uppsala CHC nurse 3yr screen • Parent language questionnaire • Language comprehension & production, observation of child’s level of cooperation • Direct child assessment & parent report • Uppsala screening • Clinical examination	• N = 2237 • 3 years • 4 years • 95% Swedish speakers • 5% Swedish language learners	• Severe developmental language disability • Child understands <3 of 5 questions • Child doesn’t utter 3 word sentences	• Nurse assessment of phonology and parent language questionnaire (intelligibility, grammar and non-fluency) • *Minor disability*: Phonological problems (Ph.pr.) scarcely influencing intelligibility. • (2) *Moderate disability*: Ph.pr. influencing intelligibility, Ph.pr. influencing intelligibility and grammatical problems, Ph.pr. influencing intelligibility and stuttering, grammatical problems with or without stuttering (priority was given to grammar). • (3) *Severe disability*: Ph.pr. heavily influencing intelligibility, Ph.pr. heavily influencing intelligibility and grammatical problems, Ph.pr. heavily influencing intelligibility and stuttering. Judgements of intelligibility were based on professional knowledge and consensus among the SLTs about the • specific effect of phonological deviations in the listener’s understanding. The extent of this influence varies as a function of the amount of deviation, kind of phonological substitute and the frequency of the phoneme in the language.	• **Referred** • Sens 86.4% Spec 98.2% • NPV N/R PPV 31.7% • **Diagnosed as disabled** • Sens 77.3% Spec 99.0% • NPV N/R PPV 42.5%

### Risk of bias

**Fig 2:**
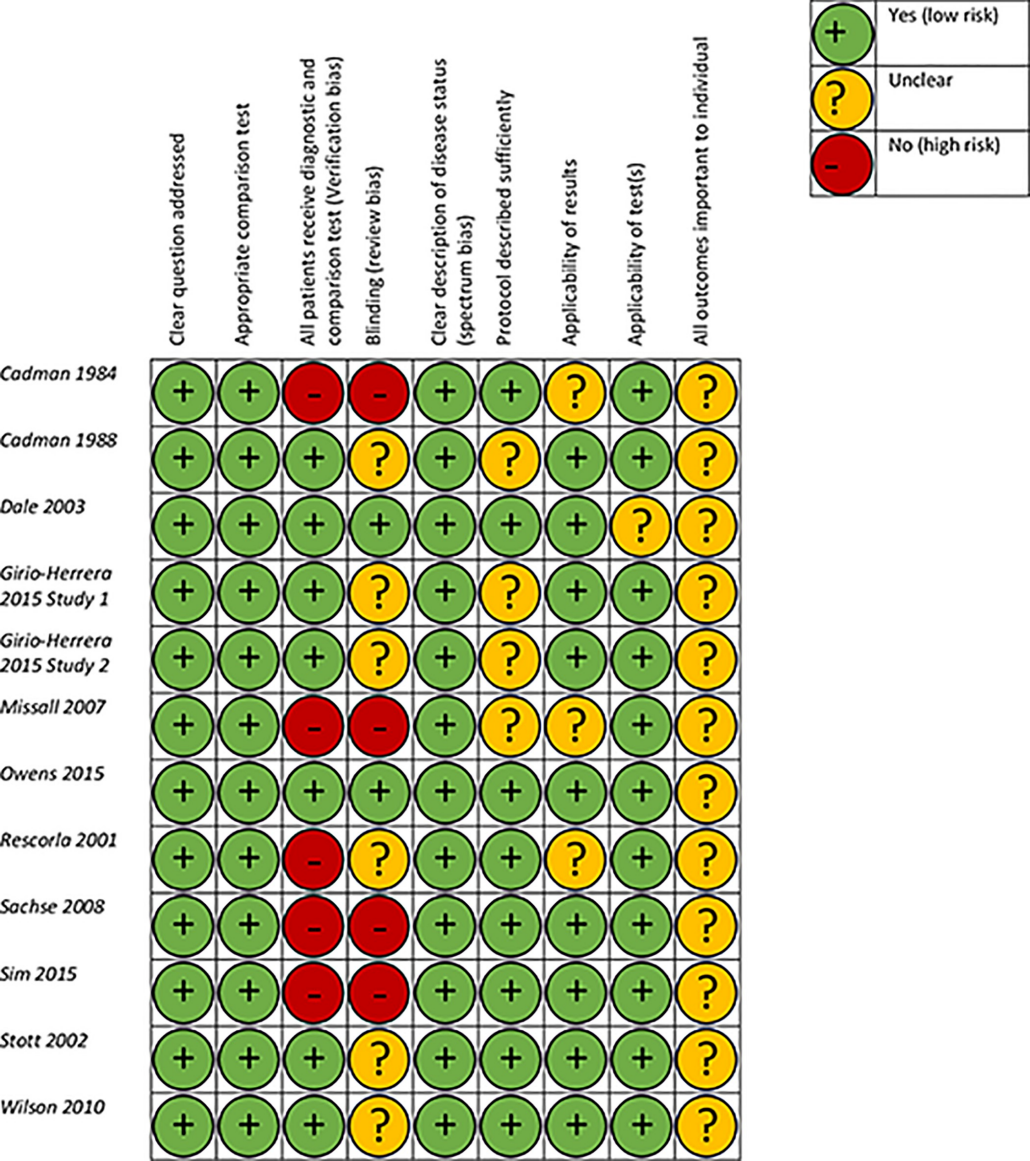
Risk of bias.

Assessment of bias data extracted using the Critical Appraisal Skills Programme (CASP) Diagnostic Checklist are presented in Fig 2.

The majority of papers had generally low risk of bias as assessed by the CASP checklist. All final papers addressed clear study questions, used appropriate comparison tests, provided clear descriptions of disease status (spectrum bias) and all but one [40] reported tests applicable to a general population setting. Risk of bias was high in the areas of verification and review bias; with five [18, 41–44] of the eleven papers reporting that all participants did not receive both the screen and diagnostic follow-up assessment and nine papers [18, 37, 41–47] reporting no or ambiguous assessor blinding to screen results.

### Risk of bias across studies

The exclusion of studies not reported in English will have introduced a degree of bias to the review as a whole, but this was judged an acceptable risk by the authors.

### Quantitative synthesis of results

**Fig 3:**
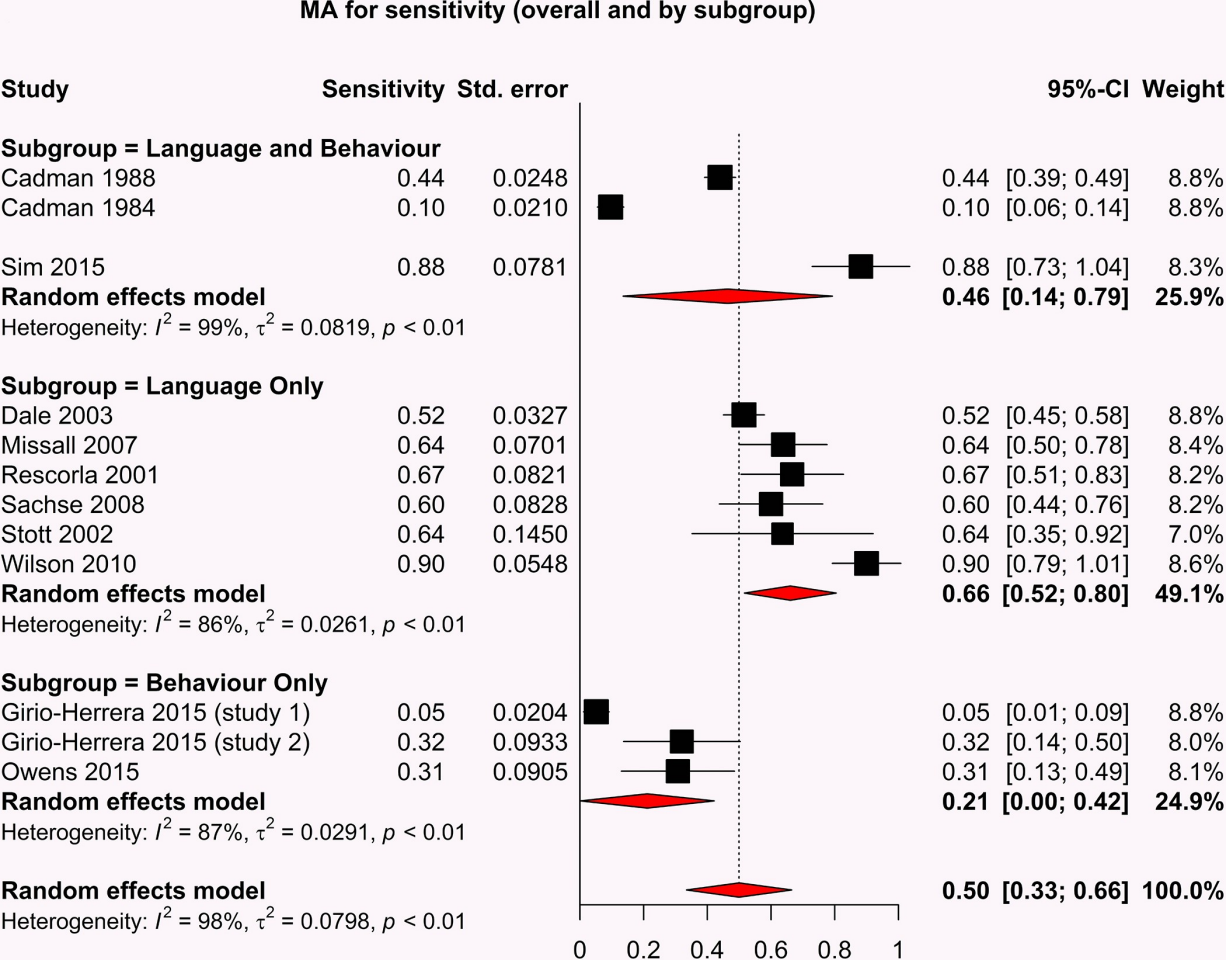
Sensitivity forest plot.

**Fig 4:**
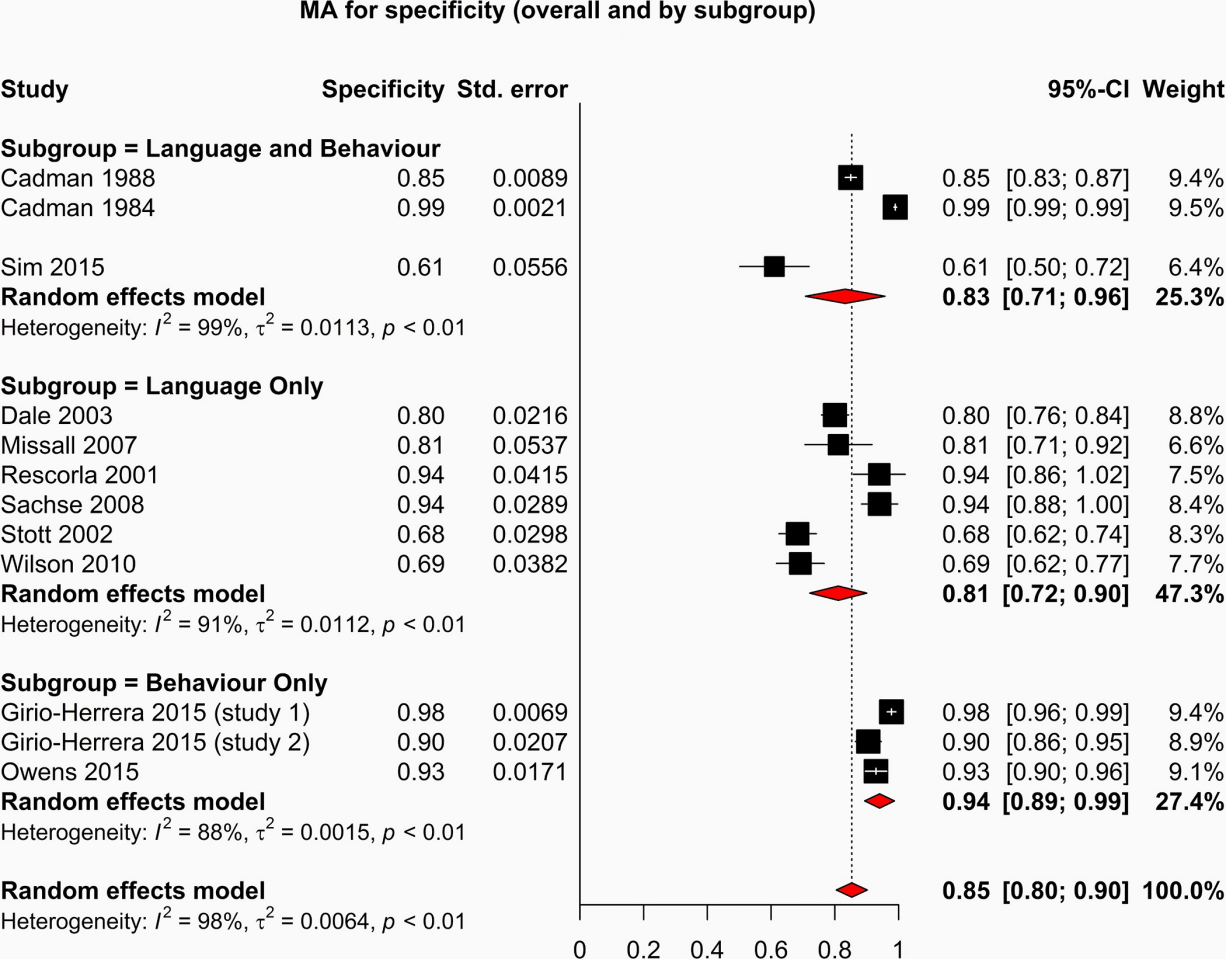
Specificity forest plot.

The forest plots depicting the sensitivity and specificity of included studies are shown in Figs 3 and 4.

Due to the variability of the outcome measure and the various tools used to assess language only, behaviour only and language and behaviour performances, we expected a high level of heterogeneity across all studies. In response to the assumption of heterogeneity, a random effects model was used to perform the meta-analysis of sensitivity, specificity and diagnostic odds ratio. The forest plots for both sensitivity and specificity indicate an overall heterogeneity (I^2^) of 98%, indicating that there are significant differences between the studies that cannot be explained by random variation.

**Fig 5:**
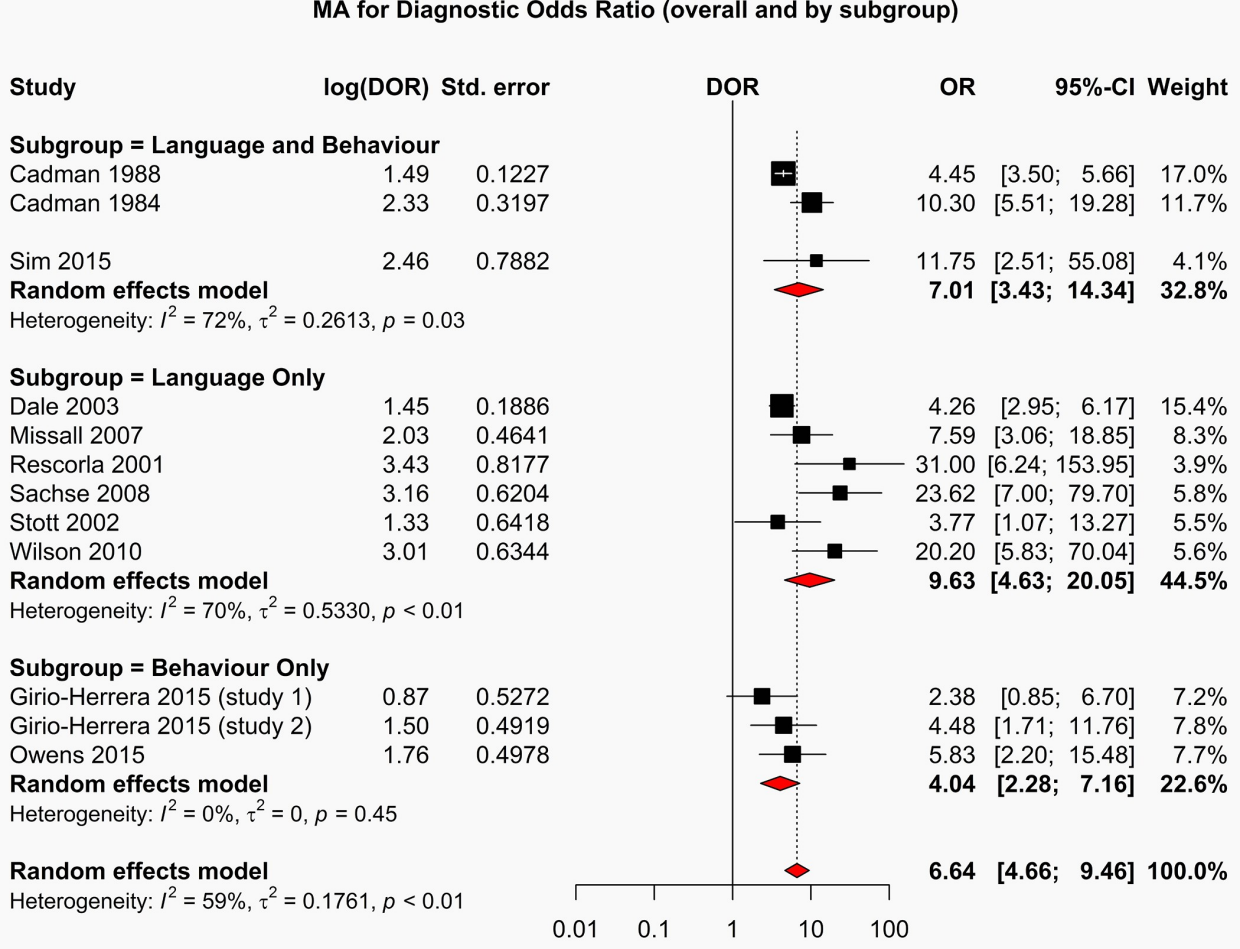
Diagnostic odds ratios forest plot.

The forest plot depicting the diagnostic odds ratios for all studies is shown in Fig 5.

The studies reporting screening tests with the highest diagnostic odds ratios are those which measure language; Rescorla et al 2001(OR: 31.00[95%CI: 6.24–153.95]); Sachse et al 2008(OR 23.62 [95%CI: 7.00 79.70]); Wilson et al 2010(OR 20.20 [95%CI: 5.83–70.04]). Studies reporting combined language and behaviour screening tools demonstrated poorer performance than language only studies but better overall performance than studies reporting behaviour only screening tools.

The performance of screening tools in descending order of diagnostic odds ratios are presented in Table 4.

**Table 4:** Performance of screening tools by Diagnostic Odds Ratio (DOR)^1^.

Screening tool	Screen cut-off	Follow-up measure (inc. cut-off)	Area assessed	Sensitivity	Specificity	NPV	PPV	DOR	Reference
**Language Development Survey (LDS)**	Delay 1 cutoff: <30 words AND no word combinations	Reynell receptive and expressive language scales Z-score less than or equal to -1.25 (10^th^ percentile)	Language	67.00	94.00	88.00	80.00	31.00 (95%CI 6.24; 153.95)	Rescorla et al 2001
**MacArthur Communicative Development Inventory (MCDI) Toddler form (ELFRA-2) Sprachentwicklungstest fur zweijahrige kinder (2.0–2.11) SETK-2**	Productive vocabulary <50 words or 50–80 words Syntax score <7, Morphology score <2	Sprachentwicklungstest fur zweijahrige kinder 3/5 (SETK-3/5) 1SD below the mean on one of three subscales	Language	61.00	94.00	95.00	56.00	23.62 (95%CI 7.00; 79.70)	Sachse et al. 2008
**Get Ready to Read! Screening Tool (GRTR)**		Test of Preschool Early Literacy (TOPEL) ELI Standard score cutoff of 90 (26^th^ percentile)	Language	90.00	69.00	38.00	97.00	20.20 (95%CI 5.83; 7-.04)	Wilson et al. 2010
**Sure Start Language Measure (SSLM)** **Strengths and Difficulties Questionnaire (SDQ)**	<32 words on SSLM >8 Total Difficulties Score SDQ	Development and Wellbeing Assessment (DAWBA) ICD-10 Psychiatric diagnosis New Reynell Developmental Language Scale (NRDLS) Comprehension or production scores 2SD below mean Griffiths Mental Development Scale-Extended Revised (GMDS-ER): General performance 2SD below mean	Language & behaviour	87.00	64.00	97.00	31.00	11.75 (95%CI 2.52; 55.08)	Sim et al. 2015
**Denver Developmental Screening Test (DDST)**		specialist referral	Language & behaviour	10.00	99.00	93.00	45.00	10.30 (95% CI 2.51; 55.08)	Cadman et al. 1984
**Early Literacy Early Growth and Development Indicators EL-EGDI’s**		Reading–Curriculum-based measurement (R-CBM) cut-off 60 words per minute	Language	64.00	81.00	72.00	74.00	7.59 (95%CI 3.06; 18.85)	Missall et al. 2007
**Strengths and Difficulties Questionnaire (SDQ)**	behaviour cut-off 4	BASC-2 BESS-teacher version Cut score for age-based *t* score of 61 or higher	Behaviour	31.00	93.00	84.00	52.00	5.83 (95%CI 2.20; 15.48)	Owens et al. 2015
**The Impairment Rating Scale (IRS)**	cut-off 2	BASC-2 BESS Teacher report behavioural and emotional problems screen T-score of 61 or greater	Behaviour	29.00	91.00	92.00	29.00	4.48 (95%CI 1.71; 11.76)	Girio-Herrera et al. 2015 (Study 2)
**Denver Developmental Screening Test (DDST) & health, behaviour & neurodevelopmental histories**	20^th^ centile	School problem–one of the following: Child still in grade 1 because of academic problems; child in a special education class; teacher rated learning problem Reading problem Lowest 10^th^ percentile on Gates-MacGinitie reading test	Language & behaviour	44.00	85.00	87.00	41.00	4.45 (95%CI 3.50; 5.66)	Cadman et al. 1988
**MacArthur Communicative Development Inventory: UK Short Form (MCDI:UKSF)** **“Displaced reference scale”** **Parent Report of Children’s Abilities (PARCA)**	10^th^ centile	Age 4yrs: MCDI inc. 48 new words; Grammar Rating Scale; Abstract Language Scale; Parental language concerns; Communicative Abnormality Scale (<29 for vocabulary, 6 for grammar, 8 for abstract language)	Language	50.00	67.30	60.50	58.10	4.26 (95%CI 2.95; 6.17)	Dale et al. 2003
**General Language Screen (GLS)**	2/11 GLS	45mths: Language function 2SD below the mean on any one of the Edinburgh Articulation Test (EAT), Reynell Developmental Language Scales (RDLS), British Picture Vocabulary Scales (BPVS).	Language	67.40	68.20	90.60	31.50	3.77 (95%CI 1.07; 13.27)	Stott et al. 2002
**The Impairment Rating Scale (IRS)**	cut-off 4	BASC-2 BESS teacher report T-score of 60 or greater on either the Externalising Problems or Internalising Problems Composites or a T-score of 40 or lower on the Adaptive Skills Composite	Behaviour	5.00	98.00	80.00	37.00	2.38 (95%CI 0.85; 6.70)	Girio-Herrera et al. 2015 (Study 1)

1 Diagnostic Odds Ratio’s (DOR) colour guide: Red = 0–9; Yellow = 10–19; Green = 20–31

### Qualitative synthesis of results

#### Predictive validity of preschool language screening tools

Six of the final eleven papers reported language-only screening tools [18, 40, 42, 43, 46, 47]. The majority (N = 4) employed a screening battery composed of multiple tools, with two of the six reporting on the use of one screening tool. The average reported administration time for these assessments was 12 minutes. The mean age of the child at initial screening assessment was 36.9 months (SD 11.2) and 43.9 months at follow-up (SD 11.4).

While most of the studies utilised the screening tool as a stand-alone measure, three studies recommended the screens would be most suited as a first step in a two-step screening process [40, 46, 47]. Four of the six final studies used parent-report measures [18, 40, 43, 46] and two used direct child-assessment [42, 47]; respondent effects on predictive validity will be discussed subsequently.

The study reporting the strongest overall predictive validity and diagnostic odds ratio was presented by Rescorla et al. 2001 [18] using the Language Development Survey (LDS) at mean age 24.7 months and the Reynell Receptive and Expressive Language Scales at mean age 25.2 months. The Language Development Study is a parent report of vocabulary and word combinations, specifically designed as a screening tool for identifying toddlers with early language delay. The authors conducted validity analyses using three different delay criteria; Delay 1 <30 words AND no word combinations; Delay 2 <30 words OR no word combinations; Delay 3 <50 words OR no word combinations. Criteria for diagnosis of expressive language delay at follow-up was a Z-score less than or equal to -1.25 on the Reynell assessment, equivalent to the lowest decile. Use of the most stringent criteria (Delay 1 <30 words AND no word combinations) provided the strongest predictive validity data; sensitivity 67%, specificity 94%, NPV 88% and PPV 80%. Overall predictive validity decreased as cut-off criteria became more inclusive (though predictably, sensitivity and NPV increased).

The second strongest overall predictive validity data was achieved by Sachse and colleagues 2008 (43) using the MacArthur Communicative Development Inventory (MCDI) Toddler form (ELFRA-2) administered at age 24 months and followed-up with the Sprachentwicklungstest für zweijährige Kinder (SETK-3/5) administered at age 37 months. The MCDI ELFRA-2 measures productive vocabulary, syntax and morphology. The ELFRA-2 parent-report predicted language delay defined by 1SD below the mean on the SETK-3/5 with 61% sensitivity, 94% specificity 95%, NPV and 56% PPV.

Missall et al. 2007 [42] reported the performance of the Early Literacy Individual Growth and Development Indicators (EL-IGDIs) which assess children’s early literacy skills, picture naming, rhyming and alliteration. The EL-IGDIs at age four years predicted reading fluency measured by a Reading-Curriculum-based measurement (R-CBM) at age six years with 64% sensitivity, 81% specificity, 72% NPV and 74% PPV.

Another study added a variant of the MacArthur Communicative Development Inventory, the UK Short Form (MCDI:UKSF) to a battery of screening tools including a 10-item Displaced Reference scale and a Parent Report of Children’s Abilities (PARCA). Administered at age two years, this screening battery predicted persistent language difficulties at age four years with 63.9% sensitivity, 70% specificity, 68.3% NPV and 65.6% PPV. Persistent language difficulties at age four was defined by a 5^th^ centile cut-off on the MCDI, grammar rating scale and abstract language rating scale.

Another screening battery approach presented by Stott and colleagues 2002 [46] used the General Language Screen (GLS) and the Developmental Profile II (DPII) administered at 36 months to predict speech and language disorders at 45 months with 67.4% sensitivity, 68.2% specificity, 90.6% NPV and 31.5% PPV. Speech and language disorders were characterised by performance 2SD below the mean on any one of the Edinburgh Articulation Test (EAT) Reynell Developmental Language Scale (RDLS), British Picture Vocabulary Scales (BPVS) and Clinical Evaluation of Language Fundamentals–Revised (CELF-R).

The study reporting the poorest predictive performance of a screening tool utilised the Individual Growth and Development Indicators (IGDIs) at initial assessment and the Test of Preschool Early Literacy (TOPEL) at follow up [47]. The IGDIs assess expressive communication, adaptive ability, motor control, social ability and cognition. Children were screened at a mean age 48.55 months and received the comparison assessment 3 months later. Predictive validity of IGDIs total score and TOPEL Definitional Vocabulary was 95% sensitivity, 6% specificity, 11% NPV, 90% PPV, AUC .71. This study reported predictive values of eight different variants of screening and follow-up assessments using both the IGDIs and the Get Ready to Read (GRTR) screen and four subscales of the TOPEL at follow-up, the most predictive combination was the GRTR and TOPEL ELI at follow up (sens 90%, spec 69%, npv 38%, ppv 97%).

The overall predictive performance of screening tools for language difficulties in pre-schoolers reported in this sample of studies is poor, with just one [18] of the six meeting the benchmark 70% sensitivity & specificity and 50% PPV recommended for developmental screening tools [48].

#### Age effects on language screening performance

Age at which children were first assessed does not appear to have a significant effect on the overall predictive performance of the language screening tools used, however the time lapse between first assessment and follow up does appear to impact on the predictive outcome.

Crosstabulation with chi-squared analysis demonstrated a significant relationship with the time interval between screen and follow up assessment and the sensitivity of the screening tools (x^2^(df) = 75; p = .05). Studies reporting a time lapse of 9 months or less [40, 42, 46, 47] between screen and follow-up broadly reported higher sensitivity data (Mean 87.34% SD 11.97) than those reporting a time lapse of 12 months or more (Mean 53.67% SD 10.87). There was no significant relationship between time interval and specificity (p = .60), PPV (.07), or NPV (p = .60). Only two of the six studies reported using receiver operating curve analysis to optimise screen performance.

#### Respondent effects on language screening performance

The final sample of studies reported here utilised either direct child assessment or parent report screening tools for language. While there is no statistically significant effect of respondent on predictive validity, it is worth noting that in all predictive outcome areas but positive predictive value, parent-report screening tools achieve higher predictive validity scores than direct child assessment.

#### Studies reporting predictive validity of language screening tools failing to meet full inclusion criteria

Four studies reporting predictive validity of preschool language screening tools but not meeting full inclusion criteria for the final sample (Table 3) are reported here in order of strength of predictive validity (those reporting strongest validity data are discussed first).

Westerlund & Sundelin [49] reported the validity of the Uppsala CHC nurse screen administered at age three years predicting severe developmental language disability diagnosed by clinical nurse examination at age four years with 77.3% sensitivity, 99% specificity and 42.5% PPV. They also reported screening validity in predicting children who would be referred for clinical examination at 86.4% sensitivity, 98.2% specificity and 31.7% PPV. This screening tool comprised direct child assessment and parent-report of language comprehension, production and observation of child’s level of cooperation. This study was rejected from the final sample as it does not report the NPV of the screening tool.

Eadie et al. [50] reported the performance of the Clinical Evaluation of Language Fundamentals (CELF-P2) administered at 4.13 years predicting language impairment 1.25SD below the mean on the CELF-4 with 64% sensitivity and 92.9% specificity. Using a more stringent cut-off of 2SD below the mean reduced sensitivity to 42.1% but improved specificity to 98.6%. This screening tool is a direct child assessment of both receptive and expressive language development. This study was rejected from the final sample as it does not report NPV or PPV and the authors had concerns over the comparative value of using two editions of the same assessment as screening and follow-up assessments.

Westerlund [51] reported further data utilising the Uppsala screening test and a parent report language questionnaire administered age four years in predicting language impairment diagnosed by a speech therapist at school start (c. age 7 years). The screen predicted severe language impairment with 12% sensitivity, 99% specificity and 43% PPV; moderate to severe impairment with 48% sensitivity, 88% specificity and 19% PPV; slight to severe impairment with 71% sensitivity, 69% specificity and 12% PPV. This study was rejected from the final sample as it does not report the negative predictive value of the screening tool.

De Koning et al [52] reported screening performance of the VTO Language Screening Instrument (VTO:LSI) administered at ages 18 and 24 months in predicting specialist service referral and language delay measured by the Dutch Parent Language Checklist (PLC); the LSI (age 3-4yrs); the LSI parents questionnaire (PQ); and Van Wiechen items at age 36 months. The VTO:LSI predicted specialist service referral with 52% sensitivity and 55% PPV; and parent-reported language delay with 24% sensitivity and 97–98% specificity. The VTO:LSI is a parent-report measure of language production, comprehension and interaction. This study was rejected from the final sample as it did not present complete predictive validity data (sensitivity, specificity, NPV & PPV) for either outcome.

The study reported by Sim et al [44] met criteria for inclusion in the final sample based on data obtained from their combined language and behavioural screening tool, but this study also reported sensitivity and specificity data for the individual language and behavioural tools utilised in the screening assessment. Using a cut-off of 31.5 out of 50 words on the Sure Start Language Measure (SSLM), screening at 30 months predicted comprehension or production difficulties identified by the New Reynell Developmental Language Scale (NRDLS) 1–2 years later with 87% sensitivity and 83% specificity.

### Predictive validity of preschool behavioural screening tools

Two of the final eleven papers reported behaviour-only screening tools [37, 53]. Both of these studies employed two concurrent screening tools and compared with a diagnostic assessment at follow-up. Both employ the Behavior Assessment System for Children–Second Edition as the gold standard comparison assessment. Results from the publication by Girio-Herrera et al. 2015 [37] are presented as two distinct studies and so for ease of understanding, results are reported separately here.

The highest combined predictive validity and diagnostic odds ratio for a behavioural screening tool comes from the Strengths and Difficulties Questionnaire (SDQ) parent-report behavioural difficulties subscale using a cut-off of 4 in predicting results from the BASC-BESS teacher report at follow-up [53].The mean age of the child at screening was 4.87 years and follow-up assessment was six months later. Results were sensitivity 31%, specificity 93%, NPV 84% and PPV 52%. The authors of this study reported validity data using both the SDQ and the Disruptive Behaviour Disorders (DBD) rating scale across a range of subscales and cut-offs.

The poorest predictive outcome from studies reporting behavioural screening tools was achieved by the SDQ parent-report emotional problems (cut-off 1) predicting the BASC-BESS teacher report six months later (sensitivity 58%, specificity 39%, NPV 79%, PPV 19%, AUC .50 95%CI (.37, .62)). This cut-point is highly inclusive and a child achieving this score would generally be considered to be within the normal range, thus explaining the particularly low positive predictive value.

Across both studies reported by Girio-Herrera et al., the Impairment Rating Scale demonstrated excellent specificity (90–98%) and NPV (80–92%) in predicting both BASC-2 and BASC-BESS teacher-reported difficulties. This parent and teacher report measure of child impairment appears to be highly accurate in identifying a subgroup of children who have difficulties and correctly classifying those who screened negative for the delay/disorder but the sensitivity and PPV are particularly low (sensitivity 5–29%, PPV 22–37%).

#### Age effects on behaviour screening performance

Age at which children were first assessed does not appear to have a significant effect on the overall predictive performance of the behaviour screening tools used, however the time lapse between first assessment and follow up does appear to impact on some elements of the predictive outcome.

Crosstabulation with chi-squared analysis demonstrated a significant relationship with the time interval between screen and follow up assessment and the NPV of the screening tools (x^2^(df) = 16; p = .044). Studies reporting a time lapse of 4 months or more [37, 53] between screen and follow-up broadly reported higher NPV data (Mean 85.54% SD 4.01) than those reporting a time lapse of 2 months or less (Mean 80% SD .00). There was no significant relationship between time interval and sensitivity, specificity, or PPV.

#### Respondent effects on behaviour screening performance

The final sample of studies reported here utilise either parent report or a combination of parent report & direct child assessment screening tools for behaviour. As with language screening tools there is no statistically significant effect of respondent on predictive validity of behaviour screening tools. Studies reporting parent-report only demonstrate higher sensitivity and PPV, and those reporting combined parent report & direct child assessment demonstrate higher specificity and NPV.

#### Studies reporting predictive validity of behaviour screening tools failing to meet full inclusion criteria

As mentioned above, the study reported by Sim et al [44] met criteria for inclusion in the final sample based on data obtained from their combined language and behavioural screening tool, but this study also reported sensitivity and specificity data for the individual language and behavioural tools utilised in the screening assessment. Using a Total Difficulties Score cut-off of 8.5 on the Strengths and Difficulties Questionnaire (SDQ), screening at 30 months predicted psychiatric disorder identified by the Development and Wellbeing Assessment (DAWBA) 1–2 years later with 68% sensitivity and 87% specificity.

#### Predictive validity of screening tools combining both language and behavioural elements

A distinct group of the final sample reported the use of a screening battery comprising both language and behavioural elements [41, 44, 45]. Two studies utilised the Denver Developmental Screening Test (DDST) allowing for holistic assessment of multiple areas of the child’s development; gross motor, language, fine motor-adaptive and personal-social development. The third study utilized a battery of screening tools assessing language & socio-emotional development [44].

The combined screening tool generating the strongest overall predictive performance was reported by Sim et al. 2015 [44]. This analysis used a combined screening tool comprising the Sure Start Language Measure (SSLM) and the Strengths and Difficulties Questionnaire (SDQ) at age 30 months, followed up by direct assessment of the child at a mean age of 47.5months using the New Reynell Developmental Language Scale (NRDLS) and the Development and Wellbeing Assessment (DAWBA). The predictive validity of this screening tool was sensitivity 87%, specificity 64%, NPV 97% and PPV 31%. The authors note that whilst the original follow-up sample over-represented screen positives, the sample was extrapolated in order to compensate for this and provide a sample representative of the whole population.

Of the two studies utilising the Denver Developmental Screening Test (DDST), the study reported by Cadman et al. 1988 reported the better predictive validity data. Screening with the DDST and a health, developmental and behavioural history at age 3.9–5.2 years predicted school problems at 6.9–8.2 years with 44% sensitivity, 85% specificity, 87% NPV and 41% PPV. A child was identified as having school problems if at least one of the following problems was present; child still in grade 1 because of academic problems; child in a special education class; teacher rated learning problem; Lowest 10^th^ percentile on Gates-MacGinitie reading test. The DDST combined with health developmental and behavioural history demonstrated better predictive validity than the DDST used alone (sensitivity 6%, specificity 99%, PPV 73%).

The study reporting the poorest predictive validity of a combined screening tool utilised the DDST administered at age 47-62months and receipt of special attention in the classroom at age 61-76months as the outcome measure [41]. This study reported 21% sensitivity (95%CI 21–22), 5% specificity (95%CI 3–7), 79% NPV (95%CI 77–80) and 62% PPV (95%CI 46–76).

#### Age effects on combined language & behaviour screening performance

Neither the age at which children were first assessed nor the time lag between screening and follow-up assessments has a significant effect on the predictive performance of the combined language & behaviour screening tools used.

#### Respondent effects on behaviour screening performance

The final sample of studies reported here utilise either direct assessment of child, parent report or a combination of parent report & direct child assessment screening tools for language & behaviour. While there is no statistically significant effect of respondent on predictive performance of the combined tools; sensitivity and NPV were higher for parent-report assessments and specificity and PPV were higher for direct child assessments.

#### Studies reporting predictive validity of combined language & behaviour screening tools failing to meet full inclusion criteria

The study reported by Fowler et al, utilising the Risk Index of School Capability (RISC) failed to meet inclusion criteria as it did not report the negative predictive value of this screening tool [54]. The study is however worth mentioning as the screening tool, developed by the authors, is unique in its incorporation of multiple risk indicators (maternal education, family history of learning problems, child’s age and gender) and direct assessment (physician rating of child attention span). Administered at age 55 months and utilising child’s failure to achieve progression to the next grade level at age 79–103 months as the outcome variable; a RISC score of 7 (out of a potential score of 11, lower scores reflecting greater risk of grade failure) or above had a sensitivity of 96%; specificity of 33%; and PPV 98%. Specificity of this screening tool improved as the cut-off became lower and therefore less inclusive (≥5 78%, (≥3 97%). This study also reported on the use of the Sprigle School Readiness Test (SSRT) (PPV 35%) and the Beery Test of Visual Motor Integration (VMI) (38%) and concluded that the combination of factors in the RISC scale was more useful than either developmental screening test in predicting early school failure.

While the screening performance of the RISC reported here is impressive, it is important to note that the outcome variable against which the predictive validity is calculated, is not a gold standard diagnostic assessment. The authors report that the use of school grade failure as an outcome was selected because of its potential impact on the psychological wellbeing of the child.

## Discussion

### Summary

One of the foremost concerns expressed in literature relating to preschool developmental screening is a lack of well validated screening tools. While there are numerous studies demonstrating the construct and concurrent [36–38] validity of preschool screening tools, there is a dearth of evidence relating to the predictive validity of these tools when used within a community setting. Predictive validity is a key criterion in determining the efficacy of a screening tool as it ensures that the tool provides not just a snapshot of how a child is developing at a specific time point but also allows some insight into the progression of their development in subsequent years.

It is with this in mind that the objective of the current review was to provide a comprehensive yet concise report on the predictive validity of screening tools, currently utilised in a community preschool setting, for the assessment of language and behaviour difficulties.

Of those studies which utilised a screening tool for language development; the best performance was achieved by Rescorla et al. 2001 (based on overall predictive validity and diagnostic odds ratio), using the Language Development Survey (LDS) at mean age 24.7 months and the Reynell Receptive and Expressive Language Scales at mean age 25.2 months. Using a cut-off of <30 words AND no word combinations predicted expressive language delay with excellent predictive validity. These validity data are certainly impressive but the reader is encouraged to note the short time lag between the screen and the follow up diagnostic assessment (one month), which would undoubtedly contribute to the predictive power of this screening tool.

The study reporting the behavioural tool with the highest overall predictive validity, and diagnostic odds ratio, was by Owens et al. 2015 using the parent-report Strengths and Difficulties Questionnaire (SDQ) at age 4.87 years and the BASC-BESS teacher-report at follow-up six months later. Using a screen cut-off of 4 on the SDQ behavioural difficulties subscale predicted social-emotional and behavioural disorders with very good predictive validity.

Of those studies reporting the use of a screening tool with both language and behavioural elements, the highest overall predictive validity and diagnostic odds ratio was reported by Sim et al., using the SDQ and SSLM at 2.5years to predict NRDLS and DAWBA diagnoses 1–2 years later. The authors state that this screening tool formed part of a universal health service contact and as such, children identified as screen positives were referred to specialist services and may have received treatment before the follow-up assessments took place thereby potentially reducing the positive predictive value of this screening tool.

When predictive validity data from all final sample studies are analysed together; language screening tools demonstrate a higher mean sensitivity of 77.7% (SD 19.31) and PPV of 66.56% (SD 26.24), than either behaviour screening tools (mean sensitivity 33.88% SD 20.70; PPV 31.38% SD 9.39) or combined language and behaviour screening tools (mean sensitivity 25.75% SD 28.20; PPV 48.50% SD 14.68). Combined language and behaviour screening tools achieve the highest mean specificity of 80.38% (SD 32.77) and NPV of 87.71% (SD 5.96) compared with language (mean specificity 55.7% SD 27.00; NPV 61.87 SD 30.60) or behaviour screening tools (mean specificity 80.13% SD 17.83; NPV 84.50% SD 4.23).

The diagnostic odds ratio analysis indicates that screening tools for language are more effective, than either screening tools for behaviour or combined language & behaviour screening tools. This could indicate that preschool language concerns are more predictive of negative outcomes at follow-up or it could be that screening tools for preschool language difficulties are more refined than those for behavioural difficulties at this age. Either way, this finding complements the growing evidence base which calls to prioritise early language skills as a primary child wellbeing indicator and an essential component of routine developmental surveillance in the early years [55].

Parent report screening tools achieve higher sensitivity, specificity and negative predictive value than direct child assessment for language development and better sensitivity and positive predictive value than a combination of parent-report and child assessment for behavioural development. This finding may seem counter-intuitive, based on previous research highlighting the inaccuracy of parent-report compared to standardized assessment [56]. However, when one considers the necessary brevity of standardized direct-child assessments (particularly screening tools) compared to the holistic perception of parent-report, it is unsurprising that the brief snapshot provided by direct-assessment will provide a less rich source of information than a parent-report measure. Furthermore; the relationship between the child and examiner would undoubtedly impact upon the child’s performance during direct assessment, again strengthening the case for parent-report assessments of early language development [57].

As for behavioural difficulties, the present study demonstrates that a combination of direct child assessment and parent-report achieves better specificity and negative predictive value than either direct child assessment or parent report alone. Similarly for combined language and behavioural difficulties, parent report and direct assessment achieve better sensitivity, positive predictive value and negative predictive value for than either parent report or direct child assessment.

### Limitations

Given the extensive yield of the literature search, we believe we have retrieved almost all of the relevant literature. Three of the eleven final studies were found through secondary source searching however, so it would appear that there may have been some studies overlooked. Studies were excluded from the final sample on the basis of language (English only included), actual reported validity data (studies excluded if missing NPV or PPV) and type of publication (conference proceedings and book chapters excluded) which may limit the results of the review somewhat. Secondary source data were sought only from the bibliographies of those studies achieving inclusion in the final sample. Studies reporting data from high-risk populations were excluded to ensure only screening tools appropriate for use in a general population setting were reported, because of this studies such as those based exclusively in deprived areas [58, 59] have not been represented in this review.

Risk of bias of the included studies introduces another possible limitation in the areas of both verification and review bias. Half of the studies reported that all participants did not receive both screen and follow-up assessments, and the majority of studies did not report whether assessor blinding occurred prior to follow-up assessment.

There is also considerable variability between studies in definition of language delay; thresholds of test positivity; respondents and differences in quality of outcome measure.

## Conclusions

The review aimed to explore some of the issues surrounding universal developmental screening of preschool aged children and report on the predictive validity of screening tools for language and behaviour difficulties, which have been utilised in a community preschool setting.

If a significant concern regarding the utilization of universal screening is the time taken to administer these tools in a community setting [60], this review presents eleven studies reporting a mean administration time of 3.36 minutes (SD 5.06). Parent-report data have also been subject to some controversy in the literature and yet there are some studies attempting to challenge this [3], this review presents studies demonstrating stronger predictive validity data from parent-report than direct child assessment.

The results demonstrate that language and behavioural concerns identified in the preschool years can be predictive of later disorders of language and socio-emotional functioning. For those studies reporting language and behaviour screening tools with the highest combined predictive validity data, sensitivity appears to be the weaker element. This lower sensitivity suggests that these tools are missing a significant proportion of screen positives; however specificity, negative predictive value and positive predictive value are consistently high for these studies indicating that those who are not at risk of delay are being correctly identified and screen results are consistent at follow-up. This finding also reflects the nature of screening performance in that there is always a trade-off between sensitivity and specificity; it may be that those developing screening tools for use in the early years have deemed it more pertinent to focus on the correct identification of those who are typically developing, at the risk of missing some of those who are not.

Evidence supporting the use of parent-report measures, particularly in identifying language difficulties, is provided here; parent-report language screening tools achieved higher sensitivity, specificity and negative predictive value than direct child assessment.

Screening tools for identifying language delay in the preschool years appear to be generally more sensitive and demonstrate stronger positive predictive value than screening tools for either behaviour alone or the combined language & behaviour screening tools. This suggests that language screening tools may identify a greater proportion of children with early delay and those identified as “at risk” continue to demonstrate difficulties at follow up assessment.

The results of this review are promising and contribute to the evidence base demonstrating the predictive validity of universal screening tools for language and behaviour concerns in preschool aged children in a community setting. Whether these are utilised as stand-alone measures in a universal primary care check-up or as part of a two-stage screening process, they can be reliably used to predict child development and guide appropriate allocation of resources. Before universal preschool screening programmes can be unconditionally supported, more work is required on the pathways from identification to intervention, and more convincing evidence is required that early intervention in a screened population is more effective than waiting until parents or teachers identify difficulties. Randomised controlled trials in hitherto unscreened populations are required to achieve this aim.

## Supporting information

S1 Fig PRISMA-DTA checklist. (DOCX)

S1 Table Extracted data items. (DOCX)
