# Supplementary material for: Predictive validity of preschool screening tools for language and behavioural difficulties: A PRISMA systematic review
Source: PLoS One. 2019 Feb 4;14(2):e0211409. doi: 10.1371/journal.pone.0211409 (PMC6361441; doi:10.1371/journal.pone.0211409)
Supplement: S1 Table — (DOCX) [file pone.0211409.s002.docx]

**S1 Table. Extracted Data Items.**

| Study design |  |
| --- | --- |
| Sample size | Screen & follow-up |
| Assessment language |  |
| Statistical analysis |  |
| Name of screening test |  |
| Areas tested |  |
| Administration time |  |
| Who administered screen |  |
| Respondent | Parent/teacher/child |
| Name of follow-up test |  |
| Child age | Screen & follow-up |
| Gender |  |
| Ethnicity |  |
| Socio-economic status | Including unit of measurement |
| Diagnostic criteria: Screen | Type of delay/disorder; Cut-off/diagnostic criteria; Rationale for criteria |
| Diagnostic criteria: at follow-up | Type of delay/disorder; Cut-off/diagnostic criteria; Rationale for criteria |
| Primary results: predictive validity data | Sensitivity/specificity/negative predictive value/positive predictive value and, if reported, area under the curve (AUC) and confidence intervals |
| Secondary results: user perspectives |  |
